# Spectrum of Cognitive Impairment in Korean ALS Patients without Known Genetic Mutations

**DOI:** 10.1371/journal.pone.0087163

**Published:** 2014-02-03

**Authors:** Seong-il Oh, Aram Park, Hee-Jin Kim, Ki-Wook Oh, Hojin Choi, Min-Jung Kwon, Chang-Seok Ki, Hee-Tae Kim, Seung Hyun Kim

**Affiliations:** 1 Department of Neurology, College of Medicine, Hanyang University, Seoul, Republic of Korea; 2 Department of Laboratory Medicine, Kangbuk Samsung Hospital, Sungkyunkwan, University School of Medicine, Seoul, Republic of Korea; 3 Department of Laboratory Medicine and Genetics, Samsung Medical Center, Sungkyunkwan University School of Medicine, Seoul, Republic of Korea; University of Florida, United States of America

## Abstract

**Background:**

Cognitive impairment is associated with a negative prognosis in amyotrophic lateral sclerosis (ALS), as well as with clinical specificity. We investigate neuropsychological function in ALS patients without known genetic mutations in a Korean tertiary clinic.

**Methods:**

Three hundred and eighteen patients were enrolled in a prospective longitudinal cohort from September 2008 to February 2012. At the time of diagnosis of sporadic ALS, we carried out genetic and comprehensive neuropsychological tests on all patients, and collected demographic and clinical characteristics. Six cognitive domains, namely executive function, attention, language, calculation, visuospatial function and memory were evaluated. ANOVA and t-tests were used to assess differences in clinical characteristics and neuropsychological parameters between sporadic ALS patients. The Kaplan-Meier method and Cox proportional hazard model were used for survival analysis.

**Results:**

One hundred and sixty-six patients were categorized into five subtypes: normal cognition (ALS pure), cognitive impairment (ALSci), behavioral impairment (ALSbi), frontotemporal dementia (ALS-FTD), and other types of dementia. Seventy patients (70/166, 42.2%) were cognitively or behaviorally impaired. Among the impaired patients, eight (8/166, 4.8%) had FTD-type dementia and one (1/166, 0.6%) was Alzheimer's disease-type. The ALS patients with cognitive impairment (ALSci) and with FTD (ALS-FTD) were more severely impaired in executive function, attention, language and memory than the cognitively intact ALS patients (ALS pure). In a survival analysis, ALSci (β = 1.925, *p* = 0.025) and ALS-FTD groups (β = 4.150, *p* = 0.019) tended to have shorter survival than the ALS pure group.

**Conclusions:**

About half of ALS patients without known genetic variation have cognitive or behavioral impairment. ALS patients with cognitive abnormalities, especially FTD, have a poorer prognosis than those without cognitive impairment. In neuropsychological profiling, executive tasks were effective in identifying cognitive impairment in the ALS patients. It would be useful for clinicians to classify ALS according to neuropsychological profiles, and screen for subtle cognitive impairment.

## Introduction

Amyotrophic lateral sclerosis (ALS) involves primarily motor system degeneration [1]–[4]. Although dominated by motor dysfunction, there is increasing evidence that ALS is a multisystem disorder which affects the extra-motor cortex, cognition, behavior and extrapyramidal system [5]–[8]. The broad clinico-pathological spectrum of ALS has been explained as due to spreading neocortical disease related to the TDP-43 proteinopathies [9].

Most patients with ALS have mild cognitive impairment with subtle executive deficits, and 5% have a clinical subtype of frontotemporal lobar degeneration (FTLD) called frontotemporal dementia (FTD) [10], [11]. Several reports have suggested that ALS patients with cognitive and behavioral impairment have a poorer prognosis than those without cognitive and behavioral impairment [5], [6]. Patients with cognitive or behavioral impairment have problems with caregiver stress, quality of life, the clinical effects of ventilator use and gastrostomies, and these also have a negative influence on survival times [12]–[15].

Despite these advances, there remains controversy regarding the issue of whether all patients with ALS show some degree of cognitive or behavioral dysfunction, or whether there are distinct groups of ALS patients with and without cognitive or behavioral changes.

To date, the range and frequency of cognitive and behavioral impairment in ALS has not been fully characterized. Also, in terms of cultural background, previous studies of cognitive impairment in ALS have mostly been carried out in the West [6], [16], [17]. Because cognitive and behavioral problems might be influenced by the cultural and linguistic background [18], cognitive and behavioral function in ALS patients needs to be assessed separately in each geographic region. However, there have been few studies of large cohorts of ALS patients with cognitive impairment in Asian countries, especially in Korea [4], [19]–[21].

Therefore we performed a cross-sectional analysis in a tertiary referral clinic in order to evaluate the variety of cognitive and behavioral dysfunctions in ALS patients without known genetic mutation in a Korean cohort and investigated the relationship between cognitive dysfunction and demographic and clinical factors.

## Materials and Methods

### ALS patients

ALS patients were recruited from ongoing prospective cohort in the Motor Neuron Disease (MND) Clinic in the Neurology Department at Hanyang University Hospital in Seoul, Korea between September 2008 and February 2012. All subjects fulfilled the revised El Escorial criteria for possible, probable, probable—laboratory-supported, or definite ALS [22]. To exclude genetic factors associated with cognitive impairment and clinical course, the patients were screened for SOD1, C9ORF72, FUS, TARDBP, ANG, OPTN as described previously [23], [24]. A total of 318 patients were enrolled. Exclusion criteria included history of other neurological conditions that could affect cognition (major stroke, traumatic brain injury, learning disability and severe active epilepsy), alcohol dependence, severe active mental illness and use of high-dose psychoactive medication [16], illiteracy and serious motor or sensory deficits that hamper the administration of neuropsychological tests [25]. ALS patients with positive results in genetic testing or by family history were excluded.

### Clinical Measures

Clinical measures included demographic data (including age, sex, level of education, site of symptoms onset, disease duration [duration from symptom onset to time of diagnosis]), ALS Functional Rating Scale-Revised (ALSFRS-R; range 0–48; normal, 48) [26], [27], breathing capacity (forced vital capacity; FVC percent predicted) and progression rate [(48-ALSFRS-R score)/disease duration (month)] [27]. This study was approved by the Institutional Review Board of Hanyang University Hospital. We obtained written informed consent to participate in genetic study and enrollment of cohort. But, we did not obtained written informed consent in the issue of analysis of neuropsychologic test data. Neuropsychologic tests were performed in routine work-up for the patients with amyotrophic lateral sclerosis and data were retrospectively analyzed without any ethical issue. This study was approved by Hanyang University Hospital in genentic study and enrollment of cohort. (cohort: HYU 2010-04-011) (genetic study: HYI-10-01)

### Participant selection

**Figure 1:**
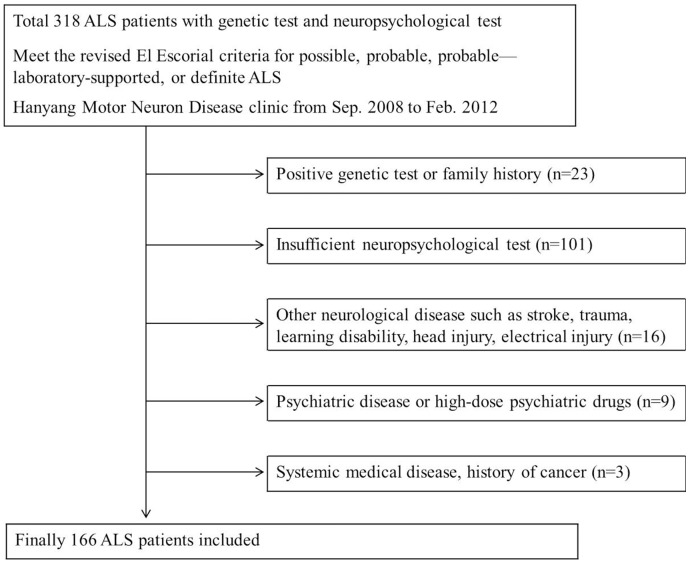
Flow chart of participant selection. Three hundred and eighteen ALS patients underwent neuropsychological testing from September 2008 to February 2012. Patients who had neurological conditions and genetic variant that could affect cognition were excluded, finally 166 patients were recruited into the study.

Three hundred and eighteen ALS patients underwent neuropsychological testing at the Hanyang University Hospital ALS clinic from September 2008 to February 2012. Of these patients, 166 patients were recruited into the study (52.2%). One hundred and fifty two patients were excluded for the following reasons: systemic medical disease (n = 3), psychiatric illness or use of psychiatric drugs (n = 9), other neurological disease (stroke, trauma, learning disability, n = 16), insufficient neuropsychological testing (n = 101), family history or genetic mutation for ALS [11 patients in SOD1 gene, one patient in FUS gene, one patient in TARDBP gene, one female patient in CAG repeat expansion of androgen receptor gene, and a notch3 mutation, 8 patients had family history which present with motor neuron disease or dementia] (n = 23) (Figure 1).

### Neuropsychological Assessment

ALS often leads to motor impairment of the upper limbs, and dysarthria. To minimize exclusions from the study (especially those in an advanced physically state), we enrolled patients who were able to complete a majority of the tests (>60% of the battery of tests), and excluded patients unable to undergo tests in at least two domains other than executive function.

Neuropsychological assessment was performed using the following standardized instruments (Table 1) [28]–[39] (1) Executive functions [contrasting program/go-no-go test, category verbal fluency test (animal item), phonemic fluency test, the Stroop test-color reading, the backward digit span test] [35], [38], (2) attention (forward digit span test) [38], (3) language (Korean version of the Boston Naming Test (K-BNT) [31], auditory comprehension test (Korean version of the Western Aphasia Battery, Yes or No question) [32], (4) calculation (3 items each of addition, subtraction, multiplication, division)) [38], (5) visuospatial function (the Rey Complex Figure Test (RCFT) [36], (6) memory including verbal memory [immediate, delayed recall, recognition tasks of the Seoul Verbal Learning Test (SVLT, Korean version of the Hopkins Verbal Learning Test)] [39], visual memory (immediate, delayed recall, and recognition tasks of RCFT) [28].

**Table 1:** Tests included in the neuropsychological assessment classified by cognitive domain.

Domain	Subdomain
Executive functions	Backward digit span
	Category verbal fluency (animal)
	Phonemic verbal fluency
	Stroop test-color reading
	Go-no-go test
	Motor impersistence
Attention	Forward digit span
Language	K-BNT
	K-WAB (Yes or No question)
Calculation	Addition, subtraction, multiplication, division (12 items)
Visuospatial function	RCFT copy
Memory	Verbal memory
	SVLT immediate, delayed recall, recognition
	Visual memory
	RCFT immediate, delayed recall, recognition

K-BNT: Korean version of the Boston naming test; K-WAB: Korean version of the Western Aphasia Battery; RCFT: Rey Complex Figure Test; SVLT: Seoul Verbal Learning Test.

We used most tasks in the comprehensive neuropsychological battery of the Seoul Neuropsychological Screening Battery (SNSB) [37], and defined cognitive impairment as one standard deviation (16th percentile) below the mean for healthy controls.

If more than one item of the verbal and visual memory test items was impaired, this was classified as abnormal memory function. If K-BNT or K-WAB (Yes or No questions) was impaired, this was classified as abnormal language function.

We used the Korean versions of the Mini-Mental State Examination (K-MMSE) [40] and Clinical Dementia Rating (CDR) [41] to evaluate patients' general cognitive impairment. Changes in behavior were assessed using the caregiver-administered Neuropsychiatric Inventory (CGA-NPI) [30]. The questionnaire included 12 neuropsychiatric domains commonly found in dementia (delusion, hallucination, agitation/aggression, depression, anxiety, euphoria, apathy, disinhibition, irritability, aberrant behavior, night behavior, appetite/eating). When two or more items had two or more points in the test this was classified as abnormal behavior. Behavior changes in terms of eating and appetite related to dysphagia due to genuine symptom of ALS were excluded.

### Patient categorization

Co-morbid frontotemporal dementia was classified as a behavioral or language variant that fulfilled the Neary criteria for frontotemporal dementia [42]. Information on behavior was obtained by direct evaluation of the patients, and via semi-structured interviews with the caregivers. Diagnosis of other co-morbid dementias was reliant on the criteria recommended by the National Institute of Neurological and Communicative Disorders and Stroke-Alzheimer's Disease and Related Disorders Association (NINCDS-ADRDA) [43].

The cohort was categorized by the recently published consensus criteria [5]. Cognitive impairment was defined as scores below the 16th percentile (one standard deviation) of the demographically corrected mean of each test on two or more tests of executive function.

### Statistical analysis

The demographic and clinical characteristics of the ALS patients were analyzed with Student's t-test. Comparisons were made using ANOVA for continuous variables and chi-square tests for categorical variables. Bonferroni's correction was applied to adjust values in cases of post hoc analysis and where multiple comparisons were undertaken. Multiple one-way ANOVAs were undertaken using two covariates: age at neuropsychologic evaluation, level of education. Survival was analyzed by the Kaplan-Meier method using the log-rank test. Multivariable analysis was performed with a Cox proportional hazard model. The level of significance was set at *p*<0.05. Statistical analyses were carried out with SPSS V.18 (SPSS Inc).

## Results

### Baseline demographics of the cohort

The mean age of the participants at diagnosis was 55.7 years (range 28.2–80.9 years, SD 10.8), and 59.0% (n = 98) were men. One hundred and twenty one patients (72.9%) had spinal onset ALS, forty three (25.9%) had bulbar-onset ALS, and of the remaining patients (n = 2, 1.2%) one had respiratory-onset and the other axial-onset ALS. Mean time from symptom onset to assessment was 17.3 month (range 4.0–49.5, SD 10.1). Mean ALSFRS-R score at baseline was 37.9 (range 21–47, SD 5.4). At the time of diagnosis, two patients (1.2%) had gastrostomy and two (1.2%) were on non-invasive ventilation of less than 4 hours per day.

### Frontotemporal dementia and co-morbid dementia

Eight patients (4.8%) fulfilled the Neary criteria for frontotemporal dementia (ALS-FTD), and one patient (0.6%) had evidence of co-morbid Alzheimer's-type dementia. The majority of ALS-FTD patients presented with behavioral changes typical of behavioral variant FTD (bv-FTD) (n = 4), and language difficulties typical of semantic dementia (SD) (n = 3). One patient presented with expressive language difficulties typical of progressive non-fluent aphasia (PNFA). There were no significant differences between patients with dementia (n = 9) and patients without dementia (n = 157) in gender, ALSFRS-R score at diagnosis, disease duration from symptom onset to examination or level of education, but bulbar onset [5/9 (55.6%) vs 38/157 (24.2%), *p* = 0.037] was more frequent, and age at neuropsychologic evaluation [62.8±8.1 vs 55.3±10.8, *p* = 0.042] was higher in the patients with dementia.

### Classification of cognitive impairments according to frontotemporal syndromes

**Figure 2:**
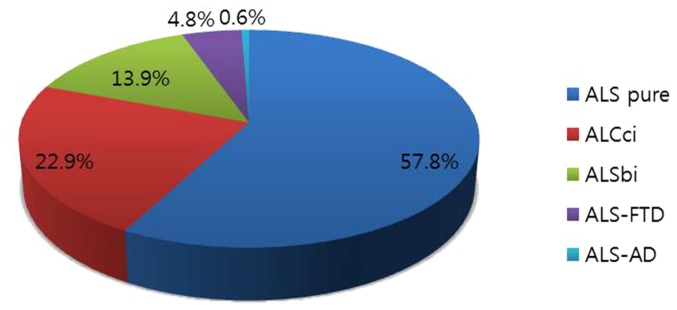
Classification of ALS in Korea according to cognitive status (n = 166). Approximately 57% of patients showed normal cognitive function and 36.8% of patients showed cognitive or behavioral impairment. Overall, approximately 5% of patients had dementia and the majority of ALS patients with dementia presented with frontotemporal dementia.

There was no evidence of dementia in one hundred and fifty seven of the patients (94.6%) according to DSM-IV, NINCDS-ADRDA or the Neary criteria [42], [43] (Figure 2). Modifying the approach used in the consensus criteria [5], we divided the non-demented ALS patients into three groups depending on the presence or absence of cognitive impairment or behavioral impairment: 1) No cognitive impairment (ALS pure) 2) Behavioral dysfunction (ALSbi) 3) Impairment in cognitive function (ALSci) : impairment in executive function or in more than two other domains of function (eg, attention, language, memory). ALS patients who had both cognitive and behavioral impairments were classified as ALSci patients. Twenty three patients (13.9%) were behaviorally impaired (ALSbi) without dementia, and 38 were cognitively impaired (ALSci), together making up 22.9% of all the patients. Considering the consensus criteria, those classificational distribution were different. If we apply the cut-off values of 2 SD in our cohort, normal cognitive or behavioral function patients are 128 (n = 128/166, 77.1%), ALSci group patients are 6 (6/166, 3.6%), ALSbi group patients are 23 (23/166, 13.9%), ALS patients with dementia are 9 patients [frontotemporal dementia: 8/166(4.9%), Alzheimer's disease: 1/166(0.6%)].

To focus on the frontotemporal syndromes in ALS patients, hereafter, one ALS patient with Alzheimer's-type dementia were excluded. Demographics and disease characteristics at baseline were compared by ANOVA or chi-square test (Table 2).

**Table 2:** Baseline demographics of ALS patients with and without frontotemporal syndromes.

	ALS pure¶	ALSbi	ALSci	ALS-FTD	Total	p-value
	(n = 96)	(n = 23)	(n = 38)	(n = 8)	(n = 165)	
Age at symptom onset, yrs	53.1±11.0	51.5±9.1	56.9±10.9	60.5±7.7	54.1±10.8	0.07
Age at neuropsychologic evaluation, yrs	54.5±11.1	53.3±8.7	58.3±10.8	61.6±7.8	55.6±10.7	0.055
Male (%)	57 (59.4)	17 (73.9)	20 (52.6)	3 (37.5)	97 (58.8)	0.233†
Education, yrs	12.1±4.3	11.0±3.1	9.3±5.0*	8.0±5.5	11.2±4.5	0.004
K-MMSE	27.0±2.4	26.8±2.9	25.7±3.1	22.9±3.0**	26.5±2.8	<0.001
CDR	0.08±0.19	0.20±0.25	0.14±0.23*	0.64±0.24	0.14±0.24	<0.001
Bulbar onset (%)	23 (24.0)	5 (21.7)	10 (26.3)	4 (50.0)	43 (25.9)	0.418†
ALSFRS-R score	39.4±4.7	34.4±5.6**	36.9±5.6	36.0±6.2	38.0±5.4	<0.001
Disease duration, mo	16.9±9.1	21.0±11.8	17.0±11.7	13.4±5.1	17.3±10.1	0.223
Progression rate	0.62±0.46	0.80±0.39	0.83±0.54	0.94±0.47	0.71±0.48	0.037

K-MMSE, Korean version of Mini Mental State Examination; CDR, Clinical Dementia Rating; Disease duration, time from symptom onset to diagnosis; Progression rate, estimated from the decline in ALS Functional Rating Scale (ALSFRS-R) score subsequent to symptom onset (48-ALSFRS-R/disease duration);

Data are means/percent ± SD. Post hoc tests were performed using Bonferroni's method.

† Chi-square tests were performed.

¶ Reference group.

* p<0.05 compared with reference group.

** p<0.01 compared with reference group.

Age at symptom onset, age at neuropsychologic evaluation, gender, bulbar onset, disease duration from symptom onset to diagnosis did not differ significantly between the four group, but level of education, K-MMSE, CDR, ALSFRS-R score at diagnosis and rate of disease progression did.

After analyzing the pure ALS group as the reference group, we compared the other groups to it: the ALSci group had a lower level of education, the ALSbi group had a lower ALSFRS-R score, and the ALS-FTD group had significantly lower K-MMSE and CDR. In progression rate, the values between groups with ANOVA and post-hoc analysis were not significantly different and age-adjusting values had no significantly difference. Although progression rate with ALSFRS-R score and neuropsychological defect had negative effect on disease prognosis, association between progression rate and neuropsychological defect were not reported clearly. These associations are unclear yet and need to be explained with the further studies.

We compared the neuropsychological profiles of the four subgroups based on their cognitive profiles. The ALS pure group and ALSci group differed with respect to impairment of the frontotemporal domain. The executive domain, including backward digit span, category verbal fluency, phonemic verbal fluency and Stroop test, and the attention domain, were significantly impaired in the ALSci group. In the attention domain examined by forward digit span, the scores of the ALS pure and ALSbi groups were similar, and the scores of the ALSci and ALS-FTD groups were both low, but significant cognitive impairment was found in the ALSci group (Table 3). In terms of language function measured by K-BNT, the ALSci and ALS-FTD groups were significantly impaired compared to the ALS pure group but the ALSbi group was not. In calculation tasks, the scores of the ALSci and ALS-FTD groups were lower than those of the ALS pure and ALSbi groups, but the differences in estimated scores between the ALSci or ALS-FTD groups and the ALS pure group were reduced after adjusting for age at neuropsychologic evaluation, level of education and ALSFRS-R score (Table S1).

**Table 3:** Cognitive domains impaired in the different groups of ALS patients.

	ALS pure¶	ALSbi	ALSci	ALS-FTD	Total	p-value
	(n = 96)	(n = 23)	(n = 38)	(n = 8)	(n = 165)	
Backward digit span	4.4±0.2	4.6±0.3	3.1±0.2**	2.7±0.6*	4.1±0.1	<0.001
Category verbal fluency	16.9±0.5	15.1±1.1	12.2±0.5**	7.4±1.1**	15.2±0.4	<0.001
Phonemic verbal fluency	29.7±1.2	24.3±1.5	16.7±1.1**	7.7±2.3**	25.1±0.9	<0.001
Stroop test-color reading	99.5±1.9	99.5±4.4	76.7±4.7**	48.8±11.1**	92.7±2.1	<0.001
Forward digit span	6.4±0.2	6.3±0.3	5.3±0.2**	5.2±0.2	6.1±0.1	0.001
K-BNT	50.9±0.8	51.5±1.6	46.7±1.2*	26.0±4.3**	48.8±0.8	<0.001
Calculation	11.4±0.2	11.5±0.2	9.7±0.5**	9.3±1.0	11.0±0.2	<0.001
RCFT	33.8±0.4	34.6±0.3	32.6±0.9	25.7±3.6**	33.3±0.4	<0.001
SVLT immediate recall	20.4±0.6	18.7±1.0	17.9±0.7	10.1±2.0**	19.2±0.4	<0.001
SVLT delayed recall	6.7±0.3	5.2±0.4	5.4±0.3*	3.3±0.8*	6.1±0.2	0.001
SVLT recognition score	21.5±0.2	21.0±0.4	20.1±0.3*	17.1±1.4**	20.9±0.2	<0.001
RCFT immediate recall	18.6±0.8	18.5±1.2	16.8±1.3	6.1±2.3**	17.8±0.6	0.008
RCFT delayed recall	18.3±0.8	17.5±1.2	16.5±1.2	6.9±2.7*	17.4±0.6	0.014
RCFT recognition score	20.5±0.2	19.9±0.4	19.7±0.3	18.4±0.9	20.1±0.2	0.044

K-BNT, Korean version of Boston naming test; RCFT, Rey complex figure test; SVLT Seoul verbal learning test.

Data are means ± SE.

Post hoc tests were performed using Bonferroni's method (<0.05).

¶ Reference group.

* p<0.05 compared with cognitively and behaviorally intact ALS patients as a reference group.

** p<0.01 compared with cognitively and behaviorally intact ALS patients as a reference group.

### The relationship between cognitive impairment and mean survival time

**Figure 3:**
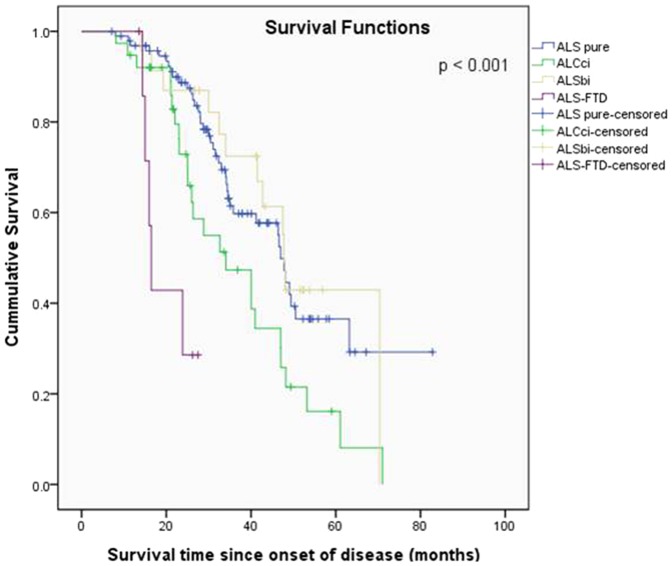
Kaplan-Meier survival curves stratified by frontotemporal syndromes. Kaplan-Meier survival plots to tracheostomy or death without tracheostomy in patients according to frontotemporal syndromes in the post-symptom onset period.

We performed a survival analysis based on the classification related to frontotemporal syndrome in the ALS patients. The median survival time of the ALS-FTD group was shorter than those of the other non-demented groups (median survival time: 16.5±0.7 months in ALS-FTD vs 47±1.1 in ALS-pure, 47.7±0.5 in ALSbi, and 34.1±6.4 in ALSci). TheALS patients without cognitive impairment, regardless of any behavioral impairment, survived longer than the ALS-FTD patients (log-rank test: *p*<0.001, Figure 3).

The following variables were included in a Cox proportional hazards model : gender, age at neuropsychologic evaluation, bulbar onset, disease duration from symptom onset to examination, progression rate (ΔFS) (rapid progression vs slow progression between two groups divided by the mean ΔFS (0.72)), FVC at diagnosis, and status of frontotemporal syndrome (intact cognition, cognitive impairment, behavioral impairment, FTD). Clinical factors including male gender (β = 1.766, *p* = 0.039), older age at neuropsychologic evaluation (β = 1.049, *p*<0.001), bulbar onset (β = 1.759, *p* = 0.048) and rapid progression (β = 7.766, *p*<0.001) were associated with shorter survival times in these ALS patients. In terms of frontotemporal syndrome, the ALS patients with cognitive impairment (ALSci) [β = 1.925, *p* = 0.025], and those with FTD (ALS-FTD) [β = 4.150, *p* = 0.019] had shorter survival times than those without cognitive or behavioral impairment, but FVC at diagnosis and ALSFRS-R score were not associated with survival times (Table 4).

**Table 4:** Cox proportional hazard model for survival with frontotemporal syndromes.

	B	SE	Sig.	Exp(β)	95.0% CI for Exp(β)	
					Lower	Upper
Male	0.568	0.275	0.039	1.766	1.03	3.027
Age at neuropsychologic evaluation	0.048	0.013	<0.001	1.049	1.023	1.077
Bulbar onset	0.565	0.285	0.048	1.759	1.006	3.075
Frontotemporal syndrome						
ALS pure (reference)			0.004			
ALSci	0.655	0.292	0.025	1.925	1.086	3.412
ALSbi	−0.454	0.358	0.205	0.635	0.315	1.282
ALS-FTD	1.423	0.607	0.019	4.15	1.263	13.632
FVC at diagnosis	0.004	0.008	0.611	1.004	0.989	1.019
ALSFRS-R score	0.035	0.026	0.184	1.036	0.983	1.091
Progression rate (>0.72)	2.05	0.303	<0.001	7.766	4.29	14.059

FVC, Forced vital capacity; ALSFRS-R score, ALS Functional Rating Scale-Revised score.

## Discussion

We documented cognitive profiles in a large prospective longitudinal cohort of ALS patients in Korea. The subtypes of demented patients in ALS consisted of the FTD and Alzheimer's disease types (n = 8 (4.8%) and n = 1(0.6%), respectively). Sixty-one patients (36.8%) had cognitive or behavioral dysfunction without dementia in our cohort. These demographic results were similar to results published previously by us and others [4]–[6], [10], [44].

Estimates of the prevalence of cognitive impairment in ALS patients vary depending on the study, the neuropsychological tests employed, sample composition, familial tendency, and genetic composition [4]–[6], [12], [45], [46]. However in most reports up to 50% of patients had cognitive impairment, with a variety of frontotemporal syndromes. Determining the spectrum of cognitive abnormalities in ALS could provide clues to their etiology, and identify the ethnic influences in different populations [16], [47].

To date, we have encountered no C9ORF72 gene mutations in our ALS cohort [23]. This mutation is the major genetic cause of ALS and FTD in Western populations, and its rarity among ALS patients in Korea could be related to the lower frequency of FTD [23], [45], [48]–[51]. That is, these ethnic genetic differences could also cause neuropsychological differences in the ALS patients with few or no C9ORF72 mutations in Korean and East Asian countries. In this study, enrolled patients have also neither TARDBP or FUS mutations. We suggest that these differences are correlated with the variation in types of dementia accompanying ALS in our cohort. In that case, unidentified genes related to frontotemporal dementia rather than C9ORF72 would be important genetic influences in Korea, and further genetic studies are needed to evaluate them.

The association between onset type and cognitive impairment in ALS is controversial. Severely impaired patients are more likely to have bulbar onset, and some studies suggest that patients with a large burden of bulbar signs are more likely to suffer neuropsychological deterioration [6], [52], but other groups found no such association [53], [54]. We detected a trend towards bulbar onset only for the most severely impaired patients such as those with ALS-FTD. These results appear to support the hypothesis that the disease process of motor neuron disease spreads gradually to nearby regions including the cortex.

The level of education in the ALS-FTD group was lower than in the ALS pure group in the present study, and higher education attainment might affect cognitive status in ALS [55].

Functional status defined by the ALSFRS-R score in the ALSbi group was also lower than in the ALS pure group. Because disease duration from symptom onset to diagnosis in the ALSbi group was longer than in the ALS pure group, these differences could be related to the different ALSFRS-R scores. Interestingly, after adjusting for disease duration, the ALSFRS-R score in the ALS pure group [39.3±0.5 (SE)] was higher than in the other three groups [36.8±0.8 (SE) in the ALSci, 35.1±1.0 (SE) in the ALSbi, 35.2±1.7 (SE) in the ALS-FTD groups], and confounding factors including disease duration and ALSFRS-R score should be carefully considered.

Our survival analysis indicated that ALS patients with cognitive impairment including ALS-FTD patients have shorter survival times than the ALS pure group. The patients with behavior impairment (ALSci) had similar survival to the ALS pure group. Abnormal behavior is considered an important manifestation in the continuum between ALS and FTD and must be taken into account in patients with ALS [13], [56]. Hu et al. [57] reported that abnormal behavior in ALS with dementia predicted shorter survival. Because all our ALS patients with FTD had abnormal behavior, we could not examine the effect of abnormal behavior on survival time in these patients. Our findings suggest that abnormal behavior in ALS patients without cognitive impairment is not related to survival time. Because the present study had insufficient power, further studies are needed of the sequential changes of abnormal behavior and the effects of the individual behaviors. Besides the neuropsychological manifestations, other currently accepted risk factors for survival in ALS patients were found in the present study. Male gender, older age at neuropsychologic evaluation, bulbar onset, and faster progression rate were associated with poor prognosis but FVC at diagnosis and ALSFRS-R score were not associated with survival time. These findings demonstrate that ALS with cognitive impairment, and especially with dementia, also has a significant negative effect on survival.

Comparisons of test scores or estimated scores in tasks related to cognitive impairment between the groups could be limited by confounding factors such as age, functional status, and educational level; hence performances in the neuropsychological tasks could help us understand the cognitive impairment. (Figure S1)

Among the types of **executive dysfunctions**, the categories verbal fluency, phonemic verbal fluency, and stroop test were abnormal in more than half the patients in the ALSci and ALS-FTD groups (Figure S1) The Stroop test had the limitation that it could not be performed in about one in three (n = 115) of the patients, because it is a long and demanding task for physically disabled ALS patients. Although the results of the backward digit span and go-no-go test were abnormal in only a small number of patients, the results in the ALSci and ALS-FTD groups were more specific than in the ALS pure group. Because the motor impersistence test was only impaired in the ALS-FTD group, it may be useful for differentiating non-demented from demented patients.

**In the attention domain**, although the forward digit span in the ALSci group was the most impaired, there was no significant difference in abnormality between the other three groups, and this task could not have value in differentiating between ALS patients. (Table S2)

**Impairment of language functions** in the ALSci and ALS-FTD groups was higher than in the ALS pure group and ALSbi groups, but less than 20% of the patients in the ALSci group were impaired in the K-BNT (18.4%) and K-WAB (7.9%) tests. Whereas the ALS-FTD patients in the present study were similarly affected in behavior and language, behavioral symptoms were more dominant than language dysfunction in the ALSci group. Although Taylor et al. [58] found that language impairment was similar in extent to executive dysfunction, the lesser prevalence of language dysfunction in the present study could be related to the use of inappropriate language tests owing to the severe bulbar dysfunction,. Therefore both language function tests may have been ineffective in identifying language dysfunction in the non-demented ALS patients, and easily applicable language tests for patients with bulbar dysfunction are needed to accurately evaluate language dysfunction.

**In the calculation tasks**, the test scores of the ALSci and ALS-FTD groups were poorer than those of the ALS pure and ALSbi groups, but the differences in the estimated scores between the ALSci or ALS-FTD group and the ALS pure group decreased after adjusting for age at neuropsychologic evaluation, level of education, ALSFRS-R score (Table S1). Therefore calculation tasks may not be useful for differentiating between groups.

**In the tests of visuospatial function** using the Rey Complex Figure Test (RCFT), the prevalence of visuospatial impairment was approximately 10% in all the groups except the ALS-FTD group. Because of the relatively small number of patients tested (n = 135) and its low prevalence in this task, the RCFT is of little value in ALS patients. These trends in terms of visuospatial dysfunction could be connected to the visual memory tasks. Although the RCFT immediate recall and RCFT delayed recall outcomes in the ALS-FTD group were more defective than in the ALS pure group, performance in the rest of the tasks was similar in all the groups. Visuospatial dysfunction was encountered in only a small number of ALS patients, and differential diagnosis using RCFT may not be useful.

**In the test of memory function**, verbal memory impairment was more prevalent than that of visual memory. As shown above, this discrepancy could be related to the difference between language dysfunction and visuospatial dysfunction. These affects are also related to semantic dementia due to left-sided dominance in ALS-FTD patients.

In the ALS pure and ALSbi groups, memory was more affected than other cognitive tasks. This memory defect could be intimately related to the poor working memory and attention, as well as the encoding of information, and to memory dysfunction itself. Also, although only one patient with dementia suffered from Alzheimer's disease-like cognitive impairment, we cannot exclude aging-related Alzheimer's disease pathology and memory impairment [59], [60] as explanations for the complex manifestations. The findings of our study should be useful in future neuroimaging and pathology studies.

The overall results of our neuropsychological profiling suggest that executive function, attention, language and memory dysfunction are more prevalent in ALS patients with cognitive impairment and those with FTD than in ALS pure patients. Using the subdomain-based classification, we demonstrated that these deficits tended to occur in the context of frontotemporal dysfunction. Backward digit span, category verbal fluency, and phonemic verbal fluency are the most efficacious and easily applicable of all the neuropsychological tasks for identifying cognitive impairment in ALS patients. Some of the ALS patients were unable to perform the comprehensive neuropsychological tests, because of physical disability, dyspnea, dysarthria, or inadequate compliance. Therefore further evaluation of easily applicable screening test for cognitive impairment in ALS patients is needed.

Our study had some limitations. First, the total number of patients enrolled in the Hanyang MND registry totaled over three hundred, but in the end only about half the registered patients participated in the study; because of motor weakness, respiratory failure and bulbar symptoms which made them unable to perform the neuropsychological tests, about half the patients (n = 111) could not be included. It is possible that the more disabled patients had more severe neuropsychological defects and that the onset of ALS with frontotemporal syndromes occurs at more advanced age, and this may have distorted our estimates of neuropsychological dysfunction in ALS patients.

Secondly, the consensus criteria for ALS-related neuropsychological dysfunction [5] use the more stringent cut-off value of two standard deviations below the mean of healthy controls, compared with the one standard deviation below the mean for healthy controls that we used for our cohort. We hypothesized that neuropsychological tests using the more lax cut-off value might identify early changes of cognitive function. Also we compensated for this procedure by defining cognitive impairment as poor performance in more than two executive function tasks or in more than two other domains. This complex combination of tasks should have helped to minimize the effect of this limitation.

Third, exclusion of ALS patients with positive genetic mutation will have altered the prevalence of frontotemporal dementia among the total ALS patients. Apart from the C9ORF72 gene, pathologic genetic mutation involving the microtubule-associated protein tau gene (MAPT), progranulin (PGRN), and valocin-containing protein (VCP) are closely associated with ALS and FTD [61], but no analysis for these genes was performed. A genetic analysis could reveal the genetic pattern and prevalence of FTD in ALS.

Fourth, in terms of longitudinal follow-up, because of rapid functional progression of bulbar and upper limb and inadequate evaluation in follow-up period, we could not explain the longitudinal change of neuropsychological function. In order to find the vulnerable neuropsychological domain, appropriate and brief test for ALS patients with rapid physical decline would be helpful for following up longitudinally.

In summary, approximately 42% of patients showed various degree of cognitive and behavioral dysfunction including dementia. That is, approximately 35%, of ALS patients suffer from cognitive or behavioral impairment, and 6% have dementia, most of which is of the frontotemporal disease-like type. In the present study, approximately 5% FTD of the non-C9ORF72-associated ALS patients had cognitive impairment and dementia in this Korean cohort - a similar rate to that in the overall ALS patient cohort. In spite of the difference in the distribution of gene mutations compared to other ethnicity, the similar findings concerning neuropsychological impairment in ALS patients encourage the performance of further analysis of unidentified genetic defects.

The survival of ALS patients with cognitive impairment or overt FTD was shorter than that of the ALS pure group. The identification of non-dementia impairment and dementia in ALS patients could lead to more ALS patients attending neurological clinics and benefiting from the rapid progress achieved, and help clinicians focus on the overall status of ALS patients.

In addition, although some of the comprehensive tests have clinical significance in identifying cognitive impairment, novel early screening method for identifying cognitive impairment need to be developed.

## Supporting Information

Figure S1 **Impaired function (%) in comprehensive neuropsychological tests among ALS patients.**
(TIF)

Table S1 **Adjusted for age, level of education, and ALSFRS-R score, estimated mean scores according to frontotemporal syndromes in multiple one-way ANOVA.** (Estimated scores, mean±SE).
(DOCX)

Table S2 **Prevalence of dysfunction in comprehensive neuropsychological tests.**
(DOCX)
