# Supplementary figures and images for: Spectrum of Cognitive Impairment in Korean ALS Patients without Known Genetic Mutations
Source: PLoS One. 2014 Feb 3;9(2):e87163. doi: 10.1371/journal.pone.0087163 (PMC3911969; doi:10.1371/journal.pone.0087163)

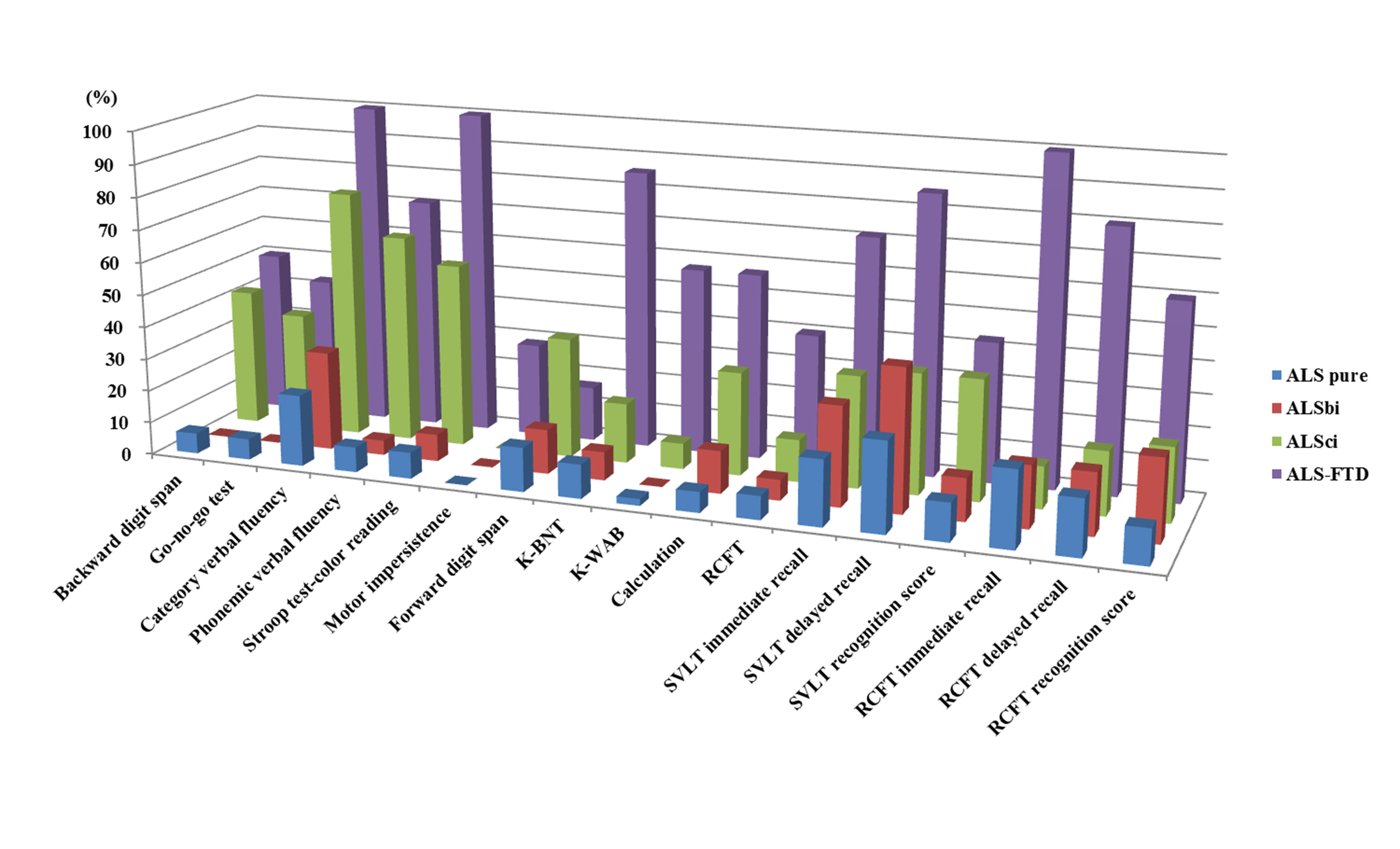

Supplement: Figure S1 — Impaired function (%) in comprehensive neuropsychological tests among ALS patients. (TIF) [file pone.0087163.s001.tif]
