# Supplementary material for: Spectrum of Cognitive Impairment in Korean ALS Patients without Known Genetic Mutations
Source: PLoS One. 2014 Feb 3;9(2):e87163. doi: 10.1371/journal.pone.0087163 (PMC3911969; doi:10.1371/journal.pone.0087163)
Supplement: Table S1 — Adjusted for age, level of education, and ALSFRS-R score, estimated mean scores according to frontotemporal syndromes in multiple one-way ANOVA. (Estimated scores, mean±SE). (DOCX) [file pone.0087163.s002.docx]

**Table S1.** Adjusted for age, level of education, and ALSFRS-R score, estimated mean scores according to frontotemporal syndromes in multiple one-way ANOVA. (Estimated scores, mean±SE)

|  | ALS pure¶ | ALSbi | ALSci | ALS-FTD | p-value |
| --- | --- | --- | --- | --- | --- |
|  | (n=96) | (n=23) | (n=38) | (n=8) |  |
| Backward digit span | 4.3±0.1 | 4.5±0.3 | 3.4±0.2 | 3.2±0.5 | <0.001 |
| Category verbal fluency | 16.6±0.4 | 14.9±0.9 | 12.9±0.7 | 8.5±1.6 | <0.001 |
| Phonemic verbal fluency | 28.6±0.9 | 24.0±1.8 | 19.2±1.4 | 9.7±3.3 | <0.001 |
| Stroop test-color reading | 95.9±1.6 | 103.8±3.9 | 82.4±2.8 | 65.8±6.1 | <0.001 |
| Forward digit span | 6.3±0.1 | 6.2±0.3 | 5.5±0.2 | 5.6±0.6 | <0.001 |
| K-BNT | 50.6±0.7 | 50.7±1.7 | 47.4±1.2 | 28.1±2.5 | <0.001 |
| Calculation | 11.5±0.2 | 11.5±0.4 | 10.7±0.3 | 9.8±0.6 | <0.001 |
| RCFT | 33.2±0.4 | 35.2±0.9 | 33.5±0.6 | 27.8±1.5 | <0.001 |
| SVLT immediate recall | 19.9±0.5 | 18.6±1.0 | 19.0±0.7 | 11.7±1.7 | <0.001 |
| SVLT delayed recall | 6.5±0.2 | 5.3±0.5 | 5.9±0.4 | 3.9±1.1 | <0.001 |
| SVLT recognition score | 21.3±0.2 | 20.8±0.4 | 20.5±0.3 | 17.7±0.7 | <0.001 |
| RCFT immediate recall | 18.1±0.7 | 18.4±1.7 | 18.1±1.2 | 7.6±3.2 | <0.001 |
| RCFT delayed recall | 17.8±0.7 | 17.4±1.6 | 17.8±1.1 | 8.3±3.1 | <0.001 |
| RCFT recognition score | 20.4±0.2 | 19.8±0.5 | 19.9±0.3 | 18.8±0.9 | 0.012 |

K-BNT, Korean version of the Boston naming test; RCFT, Rey complex figure test; SVLT Seoul verbal learning test
