# Supplementary material for: Spectrum of Cognitive Impairment in Korean ALS Patients without Known Genetic Mutations
Source: PLoS One. 2014 Feb 3;9(2):e87163. doi: 10.1371/journal.pone.0087163 (PMC3911969; doi:10.1371/journal.pone.0087163)
Supplement: Table S2 — Prevalence of dysfunction in comprehensive neuropsychological tests. (DOCX) [file pone.0087163.s003.docx]

**Table S2.** Prevalence of dysfunction in comprehensive neuropsychological tests.

| Subdomains | Abnormal results of tasks within group (%) | | | | Total (%) | p-value |
| --- | --- | --- | --- | --- | --- | --- |
| (number of tested patients) | ALS pure | ALSbi | ALSci | ALS-FTD |  |  |
| Executive function |  |  |  |  |  |  |
| Backward digit span (162) | 6.3 | 0 | 42.1 | 50 | 15.4 | <0.001 |
| Go-no-go test (157) | 6.4 | 0 | 36.1 | 42.9 | 14 | <0.001 |
| Category verbal fluency (164) | 21.9 | 30.4 | 76.3 | 100 | 39 | <0.001 |
| Phonemic verbal fluency (156) | 7.7 | 4.5 | 63.9 | 71.4 | 23.1 | <0.001 |
| Stroop test-color reading (115) | 8 | 8.3 | 56.5 | 100 | 21.7 | <0.001 |
| Motor impersistence (164) | 0 | 0 | 0 | 28.6 | 1.2 | <0.001 |
| Attention |  |  |  |  |  |  |
| Forward digit span (162) | 13.5 | 13.6 | 36.8 | 16.7 | 19.1 | 0.018 |
| Language |  |  |  |  |  |  |
| K-BNT (164) | 10.4 | 8.7 | 18.4 | 85.7 | 15.2 | <0.001 |
| K-WAB (164) | 2.1 | 0 | 7.9 | 57.1 | 5.5 | <0.001 |
| Calculation |  |  |  |  |  |  |
| Calculation (163) | 6.3 | 13 | 31.6 | 57.1 | 15.3 | <0.001 |
| Visuospatial function |  |  |  |  |  |  |
| RCFT (135) | 7.2 | 6.3 | 12.9 | 40 | 9.6 | 0.092 |
| Verbal memory |  |  |  |  |  |  |
| SVLT immediate recall (163) | 20 | 30.4 | 34.2 | 71.4 | 27 | 0.015 |
| SVLT delayed recall (163) | 27.4 | 43.5 | 36.8 | 85.7 | 34.4 | 0.011 |
| SVLT recognition score (163) | 11.6 | 13 | 36.8 | 42.9 | 19 | 0.002 |
| Visual memory |  |  |  |  |  |  |
| RCFT immediate recall (134) | 23.2 | 18.8 | 12.9 | 100 | 23.1 | 0.008 |
| RCFT delayed recall (134) | 17.1 | 18.8 | 19.4 | 80 | 20.1 | 0.014 |
| RCFT recognition score (135) | 10.8 | 25 | 22.6 | 60 | 17 | 0.044 |

Chi-square tests were performed.
